# PyroClean: Denoising Pyrosequences from Protein-Coding Amplicons for the Recovery of Interspecific and Intraspecific Genetic Variation

**DOI:** 10.1371/journal.pone.0057615

**Published:** 2013-03-01

**Authors:** Ricardo Ramirez-Gonzalez, Douglas W. Yu, Catharine Bruce, Darren Heavens, Mario Caccamo, Brent C. Emerson

**Affiliations:** 1 The Genome Analysis Centre, Norwich Research Park, Norwich, United Kingdom; 2 Ecology, Conservation, and Environment Center, State Key Laboratory of Genetic Resources and Evolution, Kunming Institute of Zoology, Chinese Academy of Sciences, Kunming, People’s Republic of China; 3 School of Biological Sciences, University of East Anglia, Norwich Research Park, Norwich, United Kingdom; The Scripps Research Institute, United States of America

## Abstract

High-throughput parallel sequencing is a powerful tool for the quantification of microbial diversity through the amplification of nuclear ribosomal gene regions. Recent work has extended this approach to the quantification of diversity within otherwise difficult-to-study metazoan groups. However, nuclear ribosomal genes present both analytical challenges and practical limitations that are a consequence of the mutational properties of nuclear ribosomal genes. Here we exploit useful properties of protein-coding genes for cross-species amplification and denoising of 454 flowgrams. We first use experimental mixtures of species from the class Collembola to amplify and pyrosequence the 5′ region of the COI barcode, and we implement a new algorithm called PyroClean for the denoising of Roche GS FLX pyrosequences. Using parameter values from the analysis of experimental mixtures, we then analyse two communities sampled from field sites on the island of Tenerife. Cross-species amplification success of target mitochondrial sequences in experimental species mixtures is high; however, there is little relationship between template DNA concentrations and pyrosequencing read abundance. Homopolymer error correction and filtering against a consensus reference sequence reduced the volume of unique sequences to approximately 5% of the original unique raw reads. Filtering of remaining non-target sequences attributed to PCR error, sequencing error, or numts further reduced unique sequence volume to 0.8% of the original raw reads. PyroClean reduces or eliminates the need for an additional, time-consuming step to cluster reads into Operational Taxonomic Units, which facilitates the detection of intraspecific DNA sequence variation. PyroCleaned sequence data from field sites in Tenerife demonstrate the utility of our approach for quantifying evolutionary diversity and its spatial structure. Comparison of our sequence data to public databases reveals that we are able to successfully recover both interspecific and intraspecific sequence diversity.

## Introduction

Challenges for quantifying microbial and meiofaunal community diversity have seen high-throughput parallel (HTP) sequencing used as a direct approach to the problem (e.g. [1], [2], [3], [4], [5], [6], [7]). The gene of choice for amplicon HTP sequencing of bacteria has been the 16S small-subunit ribosomal gene, due to its ubiquitous presence in microbes, and conserved sequence motifs facilitating cross-species amplification [1], [2], [3], [4]. Recent investigations in non-bacterial communities such as fungi [4], protists [8], [9], marine meiofaunal elements [10], [11] and nematodes [12] have also taken advantage of similar properties within nuclear ribosomal RNA (rRNA) for amplicon HTP sequencing.

The extent to which nuclear rRNA remains an optimal marker as research efforts extend from prokaryotic to eukaryotic systems has recently been questioned [13], [14], [15]. A consideration of important amplicon criteria for diversity analyses utilising HTP sequencing, and the relative merits of mitochondrial DNA (mtDNA) genes, particularly protein-coding genes, and specifically the Cytochrome Oxidase subunit 1 (COI) gene, suggests there are distinct advantages over nuclear rRNA. Amplicons should effectively be single copy and present in all taxa of interest, and with the exception of a few protozoa, these criteria are met by mitochondrial DNA. For cross-taxon utility, primers should be capable of amplifying an amplicon across all taxa. Like nuclear rRNA genes, mitochondrial 12S and 16S genes satisfy this criterion by the presence of highly conserved regions that span more variable regions. However, the evolutionary properties of protein-coding genes within the mitochondrial genome also provide the potential for universal cross-taxon amplification [16], and several recent studies demonstrate the potential for mtDNA COI primers to capture sample diversity for pyrosequencing [15], [17], [18]. Recently developed bioinformatic tools [19], [20] facilitate the design of degenerate primer pairs for improved cross-taxon amplification (e.g. [15]). In addition to these criteria, it is desirable to maximise the capture of taxonomic diversity within a focal group, and the faster evolutionary substitution rate of the mtDNA genome over nuclear rRNA genes provides for greatly enhanced taxonomic resolution.

Two additional desirable criteria for an amplicon are a taxonomic reference library for the assignment of taxonomic identity to amplicon HTP sequences, and the minimisation of artefact sequences from the HTP parallel sequencing process, a phenomenon that if uncorrected may result in spurious estimates of diversity [21], [22]. Regarding the first criterion, mtDNA gene sequences are well represented on public databases, and in particular the 5′ end of the COI gene is the focal region for the Barcode of Life Data System (BOLD) [23], which is currently represented by well over 1 million specimens with barcode sequences. The current platform of choice for amplicon HTP parallel sequencing is Roche’s GS FLX (‘454’) System (and the GS Junior version), but the noise component associated with flowgrams can confound accurate diversity assessment [11], a factor that has encouraged the development of bioinformatic tools to control for such noise (see [14] for a review). Protein-coding genes offer a potential advantage over rRNA genes for noise reduction, due to their differing functional constraints. While rRNA genes may naturally exhibit insertions or deletions (indels), the 5′ region of the mtDNA COI gene does not, with the rare exception of amino acid insertion or deletion. Thus, while the distinction between a genuine indel, and an indel generated by a homopolymer read error may complicate the denoising of 454 sequence data for a rRNA gene, this problem does not exist for mtDNA COI. Thus, the conservative evolutionary nature of many COI amino acid residues [16] facilitates sequence alignment and homopolymer error correction (e.g. [24]).

Here we exploit properties of the mtDNA COI gene for its amplification across pooled samples of divergent eukaryote species and for the development of an algorithmic approach called PyroClean for disentangling pyrosequencing noise from true sequence diversity. Our approach differs from denoising approaches for rRNA genes (e.g. [25], [26]) by incorporating a consensus reference sequence, a development recognised as important for improved noise reduction [26]. We assess our approach using the class Collembola, an example of a diverse major eukaryotic group that is poorly understood in terms of diversity and the spatial structuring of this diversity [13], [27]. We first pyrosequenced mtDNA COI amplicons from pooled genomic DNA extracts of 27 taxa of known sequence composition to assess primer functionality, evaluate algorithm performance, and obtain parameters for the analysis of mixtures of unknown sequence composition. We then analysed community samples of Collembola species sampled from two forest sites on the island of Tenerife, comparing the performance of PyroClean against a recently described pipeline for denoising protein-coding sequence data [15].

## Materials and Methods

### Test Pools

Primers were designed to amplify a 307 base pair (bp) fragment of the mtDNA COI gene by modification of the primer LCO1490 [16] and design of a downstream primer, using available Collembola sequence data on GenBank. Primer LCO1490 was modified to have improved matching to Collembola and full degeneracy across the three 3′ codons (ColFol-for, Table 1). A conserved motif of eight amino acids was chosen as the location of the reverse primer to maximise amplicon length within the suggested size constraints for Roche 454 Titanium chemistry, while also maximising primer universality. A fully degenerate reverse primer (Col307-rev, Table 1) was designed to accommodate all possible codon variation across amino acid residues. We additionally modified primer HCO2198 [16] to improve matching to Collembola for the amplification of the full 658 bp COI barcode amplicon (ColFol-rev, Table 1).

**Table 1:** Primers, MIDs, sequence formats and consensus reference sequence used in this study.

Primer	Sequence (5′ - 3′)
ColFol-for	TTTCAACAAATCATAARGAYATYGG
*ColFol-rev*	TAAACTTCNGGRTGNCCAAAAAATCA
*454-ColFol-for*	Adaptor A+MID+TTTCAACAAATCATAARGAYATYGG
*454-Col307-rev*	Adaptor B+CANCCNGTNCCNGCNCCNCTYTC
*Raw 454 sequence after demultiplexing*	>GGQCYR401C2J7Z length = 354 xy = 1142_0845 region = 1 run = R_2010_05_04_08_38_41_ TTTCAACAAATCATAAGG ATATTGGAACAATATATCTAATACTAGGATCCTGATCAGCTTTTATAGGGACTGCTTTTAGTATCCTGATCCGTATAGAACTAGGCCAACCTGGGACCCTGATTGGAAATGATCAAATCTACAATGTTATGGTGACTGCTCATGCTTTTTGTAATAATTTTCTTTATAGTTATACCAATTATGATTGGAGGGTTTGGGAATTGATTAGTCCCCCTAATAATTGGGGCTCCTGATATAGCCTTCCCACGTATAAATAATATAAGTTTCTGATTACTCCCCCCTTCCCTTACCTTATTAGTCGCGGGAGGTTTAGTAGAAAGAGCGGCAGGAACAGGA
*Unique sequence output from UniqueSequence.pl*	>GGQCYR401C2J7Z_1 ATATTGGAACAATATATCTAATACTAGGATCCTGATCAGCTTTTATAGGGACTGCTTTTAGTATCCTGATCCGTATAGAACTAGGCCAACCTGGGACCCTGATTGGAAATGATCAAATCTACAATGTTATGGTGACTGCTCATGCTTTTTGTAATAATTTTCTTTATAGTTATACCAATTATGATTGGAGGGTTTGGGAATTGATTAGTCCCCCTAATAATTGGGGCTCCTGATATAGCCTTCCCACGTATAAATAATATAAGTTTCTGATTACTCCCCCCTTCCCTTACCTTATTAGTCGCGGGAGGTTTAGTAGAAAGAGCGGCAGGAACAGGA
*PyroClean output sequence*	>Seq1_2343 ATATTGGAACAATATATCTAATACTAGGATCCTGATCAGCTTTTATAGGGACTGCTTTTAGTATCCTGATCCGTATAGAACTAGGCCAACCTGGGACCCTGATTGGAAATGATCAAATCTACAATGTTATGGTGACTGCTCATGCTTTTTGTAATAATTTTCTTTATAGTTATACCAATTATGATTGGAGGGTTTGGGAATTGATTAGTCCCCCTAATAATTGGGGCTCCTGATATAGCCTTCCCACGTATAAATAATATAAGTTTCTGATTACTCCCCCCTTCCCTTACCTTATTAGTCGCGGGAGGTTTAGT
*Consensus reference sequence*	>EMBOSS_001 NHNNNNNTNNNTNNWNHTNKSNNNNNKNNNNNSNHYNNYNGGNDYNDNNYTNARNNYNNNNNTNNSNNNNRANNTNRSNVRNNYNRGNNNNNWNNTNRRNNRNGANCANVYNTANAAYRYNNYDRTNACNKCNCANGCNKTYDYNATRATNTTYTTYRYDGTNAKNCCNNTHWTRVTHGGNGGNHTHGGNAANTKRHTNVTNCCNNTNATRVTNRRNKCNSCNGAYATNKCNTTNCCNCGNHTNANNAAYHTRAGNTTYTGRYTNYTNCCNCCNDSNHTNNNNNTNNTNNBNNNNRGNDSNNYNDBNNANDNNGRNDNNGGNACNGGNTGRNNNNYNTAYCCNCCNNTNKCNDVNNNNNYNDBNCANNNNGGNNBNDSNRTNGANNTNDNNATYTTYWSNYTNCANYYNRCNGGNRYNNSNTMNATYYTNGGNGCNRTNARYTTYANNWSNWCNDBHDDNNAYATNNRNNNNNNNNNNNTNNNNTGRRANNDNNYNHBNYTNYTNNBNTGNDSNRTNHWHNTNACNDCNDYHYTNYTNBYNNYNDSNHTNCCNKTNNTNNNNGGNGCNRTNWCNATRYTNNTNWYNGAYCGNAANNTNAANNCNDSNTTYTTYNNNCCNDSNGGNGGNGNNGANYMNRWHYTNTWNCANCNYHWNNYY

ColFol-for ColFol-rev are the Sanger primers. 454-ColFol-for and 454-Col307-rev are the primers for mass amplification. Adaptors A and B are used by the ‘454’ sequencer to attach individual DNA molecules to microscopic beads, for subsequent sequencing. MIDs (Multiplex Identifiers) are 7 bp sequences that allow different samples to be sequenced together on a single ‘454’ plate and then separated bioinformatically for downstream analysis. There is no MID with 454-Col307-rev because we only pyrosequenced from the forward direction. Row 5 is an example of a 454 read after demultiplexing with the Roche tools. The forward primer is underlined, and the reverse primer dashed underlined. The MID tag is removed during demultiplexing. Row 6 is an example of a sequence after processing of sequences to produce a file of unique sequences.

Twenty-seven individual Collembola representing 23 morphospecies sampled from the United Kingdom, Italy and the Canary Islands were extracted for their DNA using the Qiagen Blood and Tissue kit according to manufacturer’s instructions. Each sample was individually amplified and sequenced for the 658 bp barcode amplicon with primers ColFol-for and ColFol-rev, using biotaq polymerase and the following PCR conditions: 95°C for 2 min, 40 cycles of 95°C for 1 min; 52°C for 45 s; 72°C for 1 min; and finally 72°C for 5 min. Divergences among the 27 sequence range from 1 to 60 nucleotides for the 220 nucleotides corresponding to the PyroClean output alignment (see Results). Genomic DNA from the 27 samples were combined in known concentrations for the amplification of 307 bp of the mtDNA COI barcode region using primers 454ColFol-for and 454Col307-rev, with 454 adaptors A and B attached to the 5′ ends of the respective primers. Five different pools were constructed from the 27 genomic extracts (DH) and analysed blind (RR and BCE). The percentage representation of each of the 27 genomic extracts within each of the five pools is detailed in Table S1. For each of the 5 pools, PCRs were performed in triplicate, with each replicate having a different MID-tagged A adaptor, using biotaq polymerase and the following PCR conditions: 95°C for 2 min, 25 cycles of 95°C for 1 min; 49°C for 45 s; 72°C for 3 min; and finally 72°C for 10 min. PCR products were purified using AMPure XP beads (Beckman Coulter) and concentrations quantified using the Quant-iT hsDNA Assay Kit (Life Technology). PCRs were then normalized to 5 ng/µl and equimolar pooled, then sequenced using 1/2 of a plate of a Roche 454 GSFLX sequencing platform within The Genome Analysis Centre.

### Tenerife Forest Samples

Collembola were extracted with Tullgren funnels from soil sampled from two forest sites on the island of Tenerife, site 1 (Anaga), and site 2 (Teno). Permits for sampling were obtained from the Cabildo Insular de Tenerife. For each of the two sites, one hundred randomly sampled Collembola were combined for DNA extraction, followed by amplification of the 307 bp mtDNA COI amplicon and sequencing using the conditions described above, with the exception that the 6 PCRs were sequenced with approximately 2/5 of 1/2 of a plate within The Genome Analysis Centre (DH). The taxonomic identity of PyroCleaned sequences was assessed by (1) neighbour joining analysis with sequences from a preliminary Sanger sequence library of Tenerife Collembola diversity, and (2) submission of sequences to the BOLD Identification System [23].

### Pyrocleaning of Pyrosequences

Pyrosequencing data may contain exact sequence copies of an amplified target template, which we refer to as “target” sequences, and sequences that diverge from target sequences due to sequencing error, PCR error, pseudogenes, chimeric sequences, or contamination, which we refer to collectively as “non-target” sequences. The challenge for processing pyrosequencing data is to denoise sequences by the elimination of non-target sequences. We have designed a denoising algorithm that we call PyroClean for protein-coding gene regions that are evolutionarily conserved with regard to amino acid composition and nucleotide insertion and deletion events, as these provide three useful properties for denoising: (i) some amino-acid residues are highly conserved, (ii) nucleotide variation is biased toward the third base positions of codons, and (iii) indels are virtually nonexistent. The program involves five steps for the removal of non-target sequences and generation of an alignment, facilitating a sixth step for the manual removal of remaining insertion-deletion error, chimeric sequences and presumed numts (nuclear mitochondrial DNA – copies of cytoplasmic mitochondrial DNA sequences that have been transferred into the nuclear genome).

#### Step 1. Library splitting by MID and preparation of unique sequences file

Raw 454 data is processed with the sffinfo program within the Roche 454 software package, and the resulting sff files for each MID are converted to fasta format. Bases corresponding to the forward primer are trimmed from the fasta files. Reads within these files are then collapsed so that only unique sequences are presented, with the number of reads appended to each sequence name.

#### Step 2. Construction of a consensus reference sequence

The pyrosequencing error profile associated with the Roche GS FLX and GS Junior Systems is prone to insert or remove nucleotides in homopolymer regions, and as such, insertion or deletion read errors are quantifiable as frameshift events against a known reading frame. An informative consensus reference sequence facilitates the detection and correction of such frameshift events. *Consambig* from the EMBOSS suite [28] can be used to generate a consensus reference sequence from taxonomically relevant sequences available on public data bases, incorporating IUB/IUAPC ambiguity codes to summarise expected patterns of nucleotide variation.

#### Step 3. Correction of homopolymer read error

PyroClean uses a consensus reference sequence to provide anchor points for denoising reads generated from pyrosequencing. The algorithm proceeds by analysing reads individually, identifying insertions and deletions that are associated with a homopolymer, where a homopolymer is defined as two or more adjacent nucleotides with the same state. The user can exclude reads below a desired length and trim reads to a desired maximum length.

#### Insertion error

For each read, homopolymer insertions events are assessed in sequence proceeding from the 5′ end to the 3′ of a read, using an alignment score to the consensus reference sequence that incorporates matching to IUPAC ambiguity codes. Each base, *b_i_*, in a pyrosequence read is scored by counting the number of mismatches to the consensus reference sequence, *m_i_*, from *b_i_* to *b_last_*. Homopolymer insertions are then evaluated for each base that is a mismatch by identifying the preceding homopolymer, *b_i−n_*, and recalculating the alignment score after the removal of a single homopolymer nucleotide. If *m_i−n_* improves, the homopolymer nucleotide is removed.

#### Deletion error

In an analogous way for the detection and elimination of insertion events, homopolymer deletion events are detected and corrected when the number of mismatches *m_i_* is reduced by introducing a gap between the homopolymer region before *b_i_* up to the next base in the consensus reference sequence which is non-ambiguous. The homopolymer is extended if the extension is consistent with the consensus reference sequence. If an extendable homopolymer is not found, the code from the consensus sequence is inserted before the mismatch *b_i_*.

#### Insertion-deletion compensation

The algorithm also includes a routine to deal with what we refer to as indel compensation – insertion events followed by deletion events (or vice versa) within a read, resulting in mismatching to the consensus reference sequence only between the insertion and deletion. Such local misalignment is identified as a region *b_[i-j]_* where mismatching to the consensus reference sequence involves more than one base position within *b_[i-j]_*, but where there is no more than one mismatched base position either upstream from *b_i_* or downstream from *b_j_*. Within a region *b_[i-j]_*, a homopolymer nucleotide is removed from *b_i_* and a gap is added at *b_j_* (or vice versa). To fill gaps, the two nucleotides flanking the gap are evaluated. If the gap can be replaced with one of the two nucleotide states to produce a homopolymer consistent with the consensus reference sequence, then the gap is replaced with that nucleotide state. If both flanking nucleotides can generate a homopolymer consistent with the consensus reference sequence, or none of them is a valid homopolymer, the appropriate ambiguity code is used to fill the gap.

#### Step 4. Filtering reads divergent from the consensus reference sequence

After correcting homopolymer errors, reads that are divergent from the consensus reference sequence are filtered out. This is the first step for the removal of sequence variants with nucleotide variation attributable to PCR error, sequencing error, numts, or non-target sequences. Before filtering proceeds, low frequency sequences such as singletons (those only occurring once within a homopolymer-corrected alignment) can be excluded *a priori*, based on the assumption that these are likely to be non-informative [15]. The user then defines the number of permissible mismatches between a read and the consensus reference sequence. A consensus reference sequence representing all possible polymorphism would permit a filter threshold of 0, meaning that all sequences with 1 or more nucleotide states divergent from the consensus reference sequence are removed. Decreasing the filtering stringency by a value of *x* has two consequences. It increases the probability of detecting target sequences with *x* or fewer bases not represented within the consensus reference sequence. However, decreasing the filtering stringency also reduces the efficiency of filtering out variants by the retention of non-target sequences divergent by *x* or fewer bases from a target sequence.

Because homopolymer error correction in step 3 requires anchor points downstream of a homopolymer error, the algorithm is less able to denoise the 3′ end of a matrix. This is because fewer anchor points are available as homopolymer correction proceeds toward the 3′ end of a sequence. Thus, within step 4, the user can trim the alignment to exclude remaining 3′ homopolymer error by assessing the alignment output from step 3. Trimmed sequences that are identical are then merged. If two sequences are identical except for *n* number of bases where one of the two sequences has an ambiguity code, and the ambiguity code is consistent with the corresponding base of the other sequence, the sequences are treated as identical and merged in a single sequence without the ambiguity code. The frequency counts of merged sequences are summed and appended to the name of the resulting unique sequence.

#### Step 5. Filtering reads consistent with the consensus reference sequence

A second filter can be applied to remove sequences with nucleotide variation that is consistent with the consensus reference sequence filter threshold (step 4), but attributable to PCR error, sequencing error, or numts. Within this step, sequences are clustered below a user defined divergence threshold, and sequences below a minimum frequency threshold relative to the most frequent sequence within the cluster are removed. The divergence threshold and minimum frequency parameters can be derived from the analysis of experimental mixtures. Here we use data from our experimental mixtures to parameterise the analysis of forest samples from Tenerife.

#### Step 6. Manual removal of uncorrected compensated indels, inferred numts and chimeras

Four categories of non-target sequence may persist in an alignment after step 5. The first category is uncorrected compensated indels, where the alignment error between an insertion and a deletion event within a non-target sequence is not in conflict with the consensus reference sequence. The second category is nuclear copies of mitochondrial genes (numts), divergent by more than the threshold applied within step 5. The third category is chimeric sequences. These three categories can be identified (compensated indels and chimeras), or inferred (numts) and removed (see results section). A fourth category of non-target sequence represents contaminant mitochondrial sequences from non-target genomes.

## Results

### Test Pools

All analyses were performed on the TGAC computing cluster with 400+ Intel i7 cores using LSF to dispatch jobs and run analyses in parallel. Each node had at least 2 GB of free memory, of which at execution time PyroClean never required more than 50 MB of memory, making analyses feasible for modest computational environments. Raw pyrosequence reads (ColFol-for+amplicon+ColFol-rev) were on average 343 bases long, slightly less than the expected length of 358 bases (Table 2)**.** PCRs for the three MIDs within each test pool yielded broadly similar total and unique raw sequence counts (Table 2).

**Table 2:** PyroCleaning results for mtDNA COI amplicons generated from experimental pools constructed from 27 genomic extracts from 23 Collembola species.

	Pool 1			Pool 2			Pool 3									Pool 4								Pool 5							
	MID1	MID2	MID3	MID4	MID5	MID6	MID7			MID8			MID9			MID10			MID11			MID12		MID13				MID14			MID15
Total read count																															
Raw reads	16,035	15,644	16,964	13,536	15,679	13,987	9,898		13,808			6,225		9,012			13,478			14,654			14,638			17,891			14,586		
Reads above min. length	10,843	11,931	11,608	10,333	12,220	10,738	7,587		10,628			4,060		7,087			10,272			11,518			10,848			12,538			10,391		
Step 3	10,839 [100]	11,930 [100]	11,602 [100]	10,329 [100]	12,217 [100]	10,738 [100]	7,585[100]		10,625 [100]			4,060[100]		7,087[100]			10,268 [100]			11,517 [100]			10,847 [100]			12,535 [100]			10,383 [100]		
Step 4	2,715 [25]	2,833 [24]	2,806 [24]	2,074 [20]	2,659 [22]	2,130 [20]	1,856 [24]		2,725 [26]			1,006 [25]		1,672 [24]			2,276 [22]			2,735 [24]			2,302 [21]			2,790 [22]			2,087 [20]		
Unique read count																															
Reads above min. length	9,777	10,879	10,522	9,408	11,036	9,800	6,862	9,605			3,751				6,385			9,257			10,422				9,585		11,026			9,211	
Step 3	638 [6.5]	718 [6.6]	676 [6.4]	634 [6.7]	713 [6.5]	667 [6.8]	435 [6.3]	621 [6.5]			225 [6.0]				380 [6.0]			596 [6.4]			721 [7.0]				592 [6.2]		677 [6.1]			553 [6.0]	
Step 4	169 [1.7]	190 [1.7]	173 [1.6]	149 [1.6]	166 [1.5]	125 [1.3]	124 [1.8]	161 [1.7]			65 [1.7]				89 [1.4]			138 [1.5]			156 [1.5]				110 [1.1]		153 [1.4]			114 [1.2]	

Each pool has been amplified in triplicate. Sequences were generated on 1/2 a 454 plate that generated a total of 156,315 raw reads. Raw reads had a maximum length of 534 bp and an average length of 343.4 bp. Steps 3–4 are summarised in the text. Numbers in brackets represents sequence reduction as the % of raw reads above a minimum length of 170 nucleotides.

We constructed a consensus reference sequence for Collembola (step 2) using all 1556 collembolan COI gene sequences available from EMBL/GenBank at the time (Table 1). We then implemented the PyroClean algorithm to correct homopolymer read error (step 3), ignoring sequences with a length less than 170 bases, and trimming sequences to 300 bases (slightly less than the expected length of the amplicon sequence). Across the 15 MID pools, unique sequence reads represent approximately 6.4% of unique raw reads, after homopolymer read error correction (Table 2).

**Figure 1:**
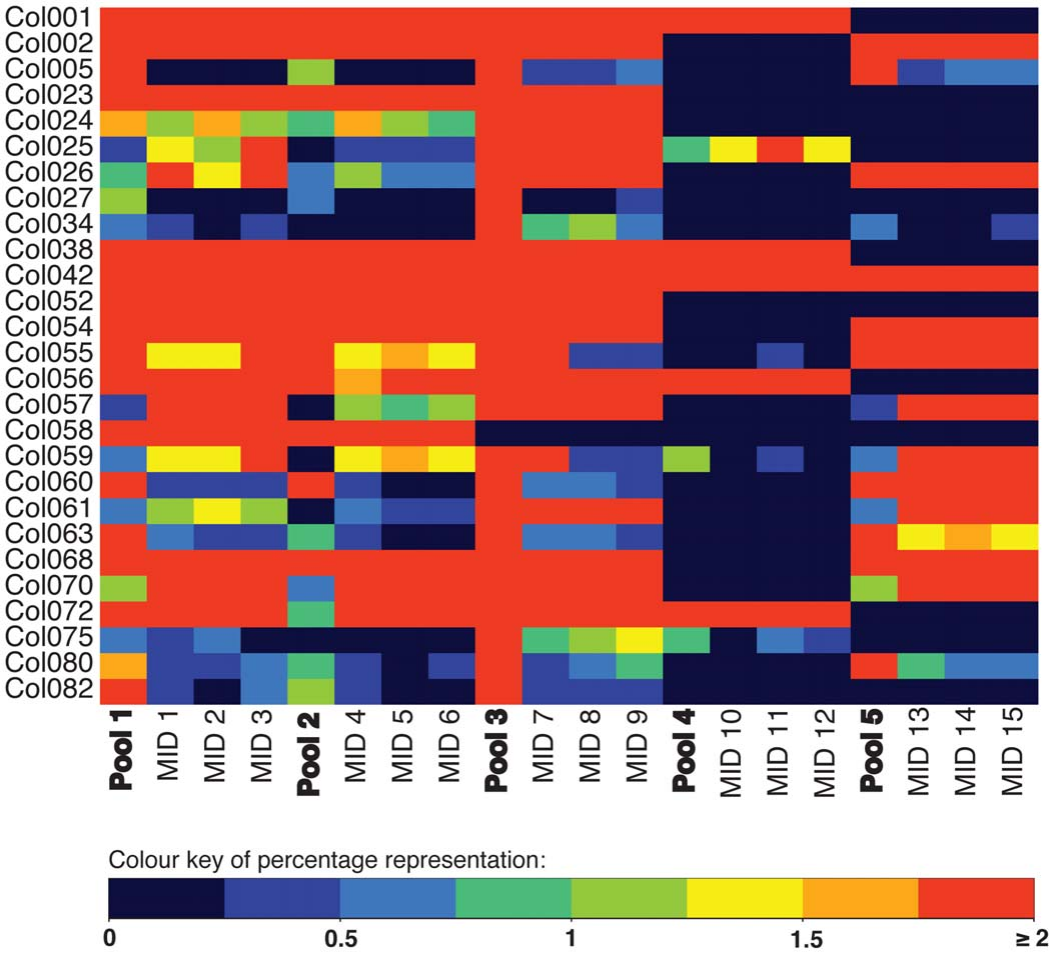
Heat map of the percentage representation of the 27 Collembola genomes within each of the 5 genomic pools, and within each of the three MID tag pools derived from each of the 5 genomic pools. For visual clarity, percentage representation of 2% or more is presented as maximum representation. See Supplementary Table 1 to see the actual percentages of each sequence found, and species names.

**Figure 2:**
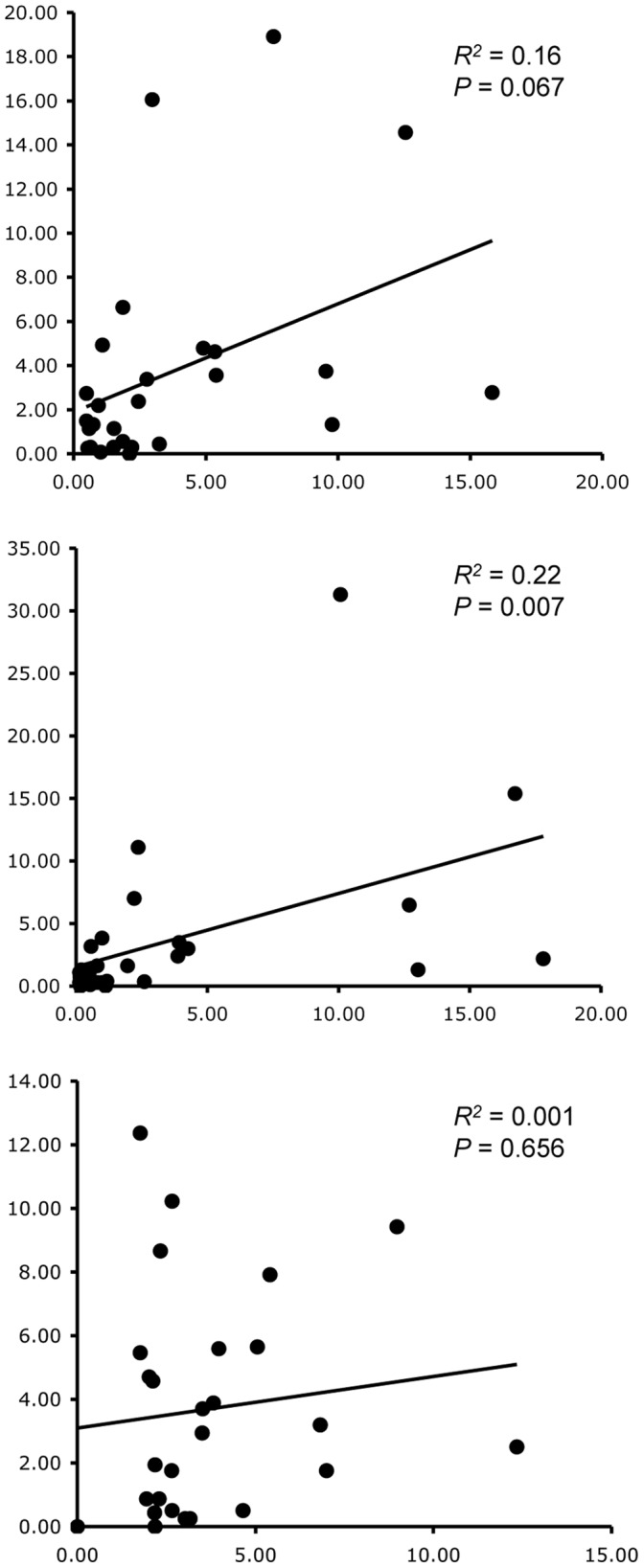
Regression analyses of the percentage of collembolan mtDNA COI sequences within an MID tag pool (*y* axis) against the percentage of genomic template from which they are derived within an experimental genomic pool (*x* axis). Data come from Table S1. The panels from top to bottom are MID1 against pool 1, MID4 against pool 2, and MID7 against pool 3.

After homopolymer read error correction, we excluded singleton sequences and then filtered remaining sequences against the consensus reference sequence (step 4) allowing 1 mismatch to the consensus reference sequence, and trimming the alignment to 220 bases after observing that homopolymer error correction was not successful beyond this point. After filtering sequences divergent from the consensus reference sequence by more than 1 nucleotide, unique sequence reads represent approximately 1.5% of unique raw reads (Table 2). Computational processing time for individual MID tag pools ranged from 13 to 27 minutes, with longer processing times for more complex genome pools. All expected target sequences were recovered for each of the 5 pools, with only a few instances of an expected target sequence not being represented in all three PCR replicates within a pool (Fig. 1, Table S1). For PCR pool 4 (MID tags 10–12), three unexpected target sequences were recovered at low frequency (Table S1), which we attribute to cross-contamination during preparation of the genomic pools. There is little relationship between the proportional representation of genomic templates and the frequency of the target sequence within an MID tag pool (Fig. 2).

In addition to target sequences, many non-target sequences remain in the alignments after filtering against the consensus reference sequence in step 4 (Table 2). Across the 15 MIDs of the 5 test pools, 80–85% of these differ by only one or two nucleotides from a target sequence, and occur at a frequency of <10% of the target sequence. These are attributable to PCR error, sequencing error, and numts that do not result in divergence from the consensus reference sequence. These sequences can be filtered out based on their low frequency and high relatedness to a target sequence, and we parameterise step 5 for the analysis of Tenerife forest samples to filter out sequences ≤1% divergent from, and <10% frequent than a related sequence in the same MID pool. Remaining non-target sequences were found to comprise uncorrected compensated indels, inferred numts, and contaminant sequences. We assessed all sequences found only in a single MID pool for chimeric origin, but none was found to be chimeric. Undetected compensated indels are evident within an alignment, as they diverge from a related sequence by a sequential run of mismatching nucleotides, facilitating their identification and removal (Fig. S1). Target sequences were in some cases associated with presumed numts - phylogenetically related, low frequency variants (<10%) of a target sequence (Fig. S2). Removal of presumed numts on the basis of low frequency relative to a related sequence in a MID pool represents a conservative approach where genuine low frequency target sequences may be lost from the data set. However, this can potentially be corrected for when larger numbers of communities are sampled (see Discussion). Three contaminant sequences (unexpected target sequences) were found in PCR pool 4 (MID tags 10–12) at low frequency (Table S1), echoing concerns over potential error from laboratory-based contamination [15].

### Tenerife Forest Samples

Amplicon PCRs for sampling sites 1 (MID 7, MID 8, MID 9) and 2 (MID 10, MID 11, MID 12) yielded broadly similar total and unique sequence counts after data processing with the sffinfo program within the Roche 454 software package (Table 3). We ran the algorithm to correct homopolymer error (step 3), excluded singletons, and filtered the first 220 nucleotides for 1 mismatch to the consensus reference sequence (step 4). Processing time took approximately 10 minutes for each MID, requiring 30 MB of RAM, and resulted in approximately 60% of the total number of raw reads. The effect on the number of unique sequences was much greater, with the volume of unique sequence reads approximately 5% of unique raw reads (Table 3). We then filtered the data by removing sequences that are both ≤1% divergent from, and <10% frequent than a related sequence from the same MID tag pool (step 5), resulting in an overall reduction of unique sequence volume to 0.5–1.3% of unique raw reads (Table 3). Filtering took less than one minute, using less than 10 MB of RAM.

**Table 3:** PyroCleaning results for mtDNA COI amplicons generated from community samples of Collembola from two forest sites on the island of Tenerife.

	Site 1			Site 2		
	MID7	MID8	MID9	MID10	MID11	MID12
Total read count						
Raw reads	11,249	11,479	9,234	7,583	11,426	11,584
Reads above min. length	7,311	7,613	6,008	5,255	7515	8,165
Step 3	7,311 [100]	7,613 [100]	6,007 [100]	5,255 [100]	7,515 [100]	8,165 [100]
Step 4	4,492 [61]	4,697 [62]	3,501 [58]	3,276 [62]	4,289 [57]	4,923 [60]
Step 5	3,644 [50]	3,815 [50]	2,879 [48]	2,844 [54]	3,540 [47]	4,114 [50]
Step 6	3,596 [49]	3,764 [49]	2,828 [47]	2,744 [52]	3,409 [45]	4,006 [49]
Unique read count						
Reads above min. length	4,742	4,962	4,067	3,356	5,234	5,473
Step 3	450 [9.5]	427 [8.6]	381 [9.3]	304 [9.1]	493 [9.4]	504 9.2]
Step 4	252 [5.3]	231 [4.7]	215 [5.3]	170 [5.1]	289 [5.5]	287 [5.2]
Step 5	27 [0.57]	27 [0.54]	30 [0.74]	44 [1.31]	49 [0.94]	46 [0.84]
Step 6	17 [0.36]	17 [0.34]	19 [0.46]	20 [0.60]	20 [0.38]	21 [0.38]

Each site has been amplified in triplicate. 62,825 raw reads were generated on 1/2 of a 454 plate from a total of 103,850 raw reads (the remainder of raw reads belonged to another experiment). Raw reads had a maximum length of 521 bp and an average length of 343 bp. Steps 3–6 are summarised in the text. Numbers in brackets represents sequence reduction as the % of raw reads above a minimum length of 170 nucleotides.

**Figure 3:**
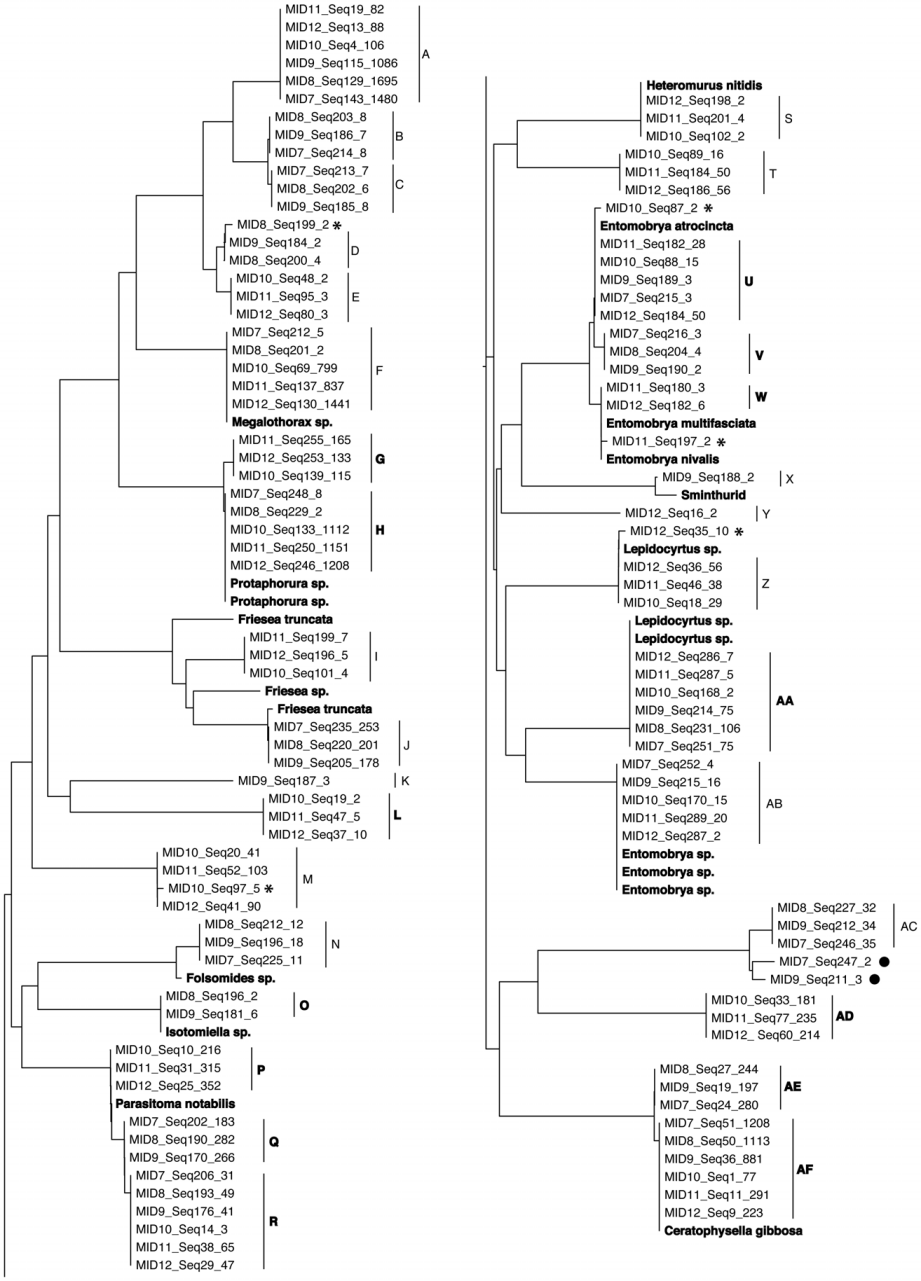
Neighbour joining tree of PyroCleaned sequences derived from two forest sampling sites in the island of Tenerife, and 21 taxonomically identified Sanger sequences samples from Tenerife (in bold). Sequence identifiers represent MID tag, the name of the sequence, and the frequency representation of the sequence. Sequences were assessed for taxonomic identity against the BOLD Identification Database, and we report closest matches for Collembola, and non-Collembola if matching is higher. Letters in bold represent sequence matches to Collembola of 99% or higher: **A** – *Tetrodontophora bielanensis* (88%), *Pelosia muscerda* (Lepidoptera, 91%), **B** – *Tetrodontophora bielanensis* (89%), *Myotis ikonnikova* (Chiroptera, 91%); **C** – *Tetrodontophora bielanensis* (89%), *Myotis ikonnikova* (Chiroptera, 91%); **D** – *Verhoeffiella sp.* (89%), *Stenopsyche* (Trichoptera, 92%), **E** – no significant match, *Stenopsyche* (Trichoptera, 94%), **F** – *Folsomia* (94%), *Ophiogomphus* (Odonata, 95%), **G** – *Protaphorura* (99%), **H** – *Protaphorura* (100%), **I** – *Xenylla humicola* (90%), *Carterocephalus silvicola* (Lepidoptera, 91%), **J** – *Bourletiella* (89%), **K** – *Lepidocyrtus violaceus* (88%), *Herona marathus* (Lepidoptera, 90%) **L** – *Tullbergia sp.* (99%), **M** – *Parisotoma notabilis* L3 (92%), **N** – *Folsomina yosii* (90%), **O** – *Isotomiella* (99%), **P** – *Parisotoma notabilis* (100%), **Q** - *Parisotoma notabilis* (100%), **R** – *Parisotoma notabilis* (100%), **S** – *Xenylla humicola* (90%), *Finlaya* (Culicidae, 91%), **T** – *Verhoeffiella* sp. (90%), **U** – *Entomobrya atrocincta* (100%), **V** - *Entomobrya atrocincta* (99%), **W** - *Entomobrya atrocincta* (100%), **X** – *Parisotoma notabilis* (94%), *Phasus* (Lepidoptera, 96%), **Y** – *Isotomurus* (92%), **Z** – *Desoria sp.* (91%), *Bathymunida nebulosa* (Decapoda, 95%), **AA** – Entomobryidae (90%), *Polytremis pellucida* (Lepidoptera, 93%), **AB** – *Isotoma sp.* (91%), *Neophylax rickeri* (Trichoptera, 92%), **AC** – *Isotomurus* (86%), Chordate (90%), **AD** – *Neanura muscorum* (100%), **AE** – *Ceratophysella sp.* (99%), **AF** – *Ceratophysella sp.* (100%). The five sequences with an asterisk are potential numts or pcr error that exceed the final filter threshold. The two sequences with a filled circle are left in as an example of probable numts.

PyroCleaning resulted in an average of 37 unique sequences within each of the 6 MID tag pools, with a total of 88 unique sequences across the 6 MID tag pools (Table 3). As in the test pool data, none of these sequences was found to be of chimeric origin. After removing uncorrected compensated indels, sequences were then subjected to a neighbour joining analysis using p-distances with MEGA5 [29]. The neighbour-joining tree revealed several clusters of closely related presumed numts (Fig. S2). Removal of these sequences reduced the average number of unique sequences within each MID tag pool to 19, representing a total of 39 unique sequences. A neighbour joining analysis reveals the 39 unique sequences to comprise 24 divergent lineages, and the taxonomic identity of 14 of these are revealed with the joint analysis of taxonomically referenced Sanger sequences, with 10 of these representing exact matches to Sanger sequences (Fig. 3). Thirty of the 37 PyroCleaned sequences were assessed for taxonomic identity against the BOLD Identification System, with 13 sequence matches of 99% identity or higher (Fig. 3).

We compared the performance of PyroClean with a recently described pipeline for denoising protein-coding sequence data [15], by analysing the same Tenerife pyrosequence data and clustering at 98% (Table 4). The pipeline of Yu *et al.* [15] resulted in an average of 607 unique denoised sequences within a MID pool prior to clustering (step 3, Table 4), compared to an average of 37 after PyroCleaning (step 5, Table 3). The efficiency of PyroClean for the removal of non-target sequences reduces the need for subsequent clustering. This means that target sequences below a clustering threshold can be recovered with PyroClean, while these would be lost with clustering. This is less important for studies that seek only to characterise species presence (e.g. [15]), but it assumes greater importance when geographic patterns of genetic relatedness within species are of interest. A useful example of this comes from *Parasitoma notabilis,* where PyroClean recovered three closely sequences, revealing shared genetic variation with populations outside the Canary Islands (see Discussion), whereas none of these sequences was recovered with the pipeline of Yu *et al.* [15]. Even after clustering at 98%, the pipeline of Yu *et al*. [15] appears to include many non-target sequences, with sequence counts close to or exceeding the number of individuals in the analysis (Table 4).

**Table 4:** Denoising of mtDNA COI amplicons generated from community samples of Collembola from two forest sites on the island of Tenerife with the pipeline of Yu *et al.* (2012).

	Site 1			Site 2		
	MID7	MID8	MID9	MID10	MID11	MID12
Total read count						
Step 1 (quality control)	10,413	10,581	8,416	7,043	10,635	10,737
Step 2 (PyNAST, 60%)	10,394	10,572	8,392	7,040	10,603	10,712
Unique read count						
Step 2 (PyNAST, 60%)	7,040	7,306	5,822	4,752	7,492	7,443
Step 2 (USEARCH)	2,021 [12]	2,032 [13]	1,621 [16]	1,452 [5]	2,255 [15]	2,147 [30]
Step 3 (MACSE)	709	719	305	152	940	817
Step 4 (DNACLUST, 99%)	580	610	267	139	805	710
Step 5 (CROP, 98%)	69	52	52	65	103	98

Steps 1–3 represent reduction of unique sequence volume by denoising, while steps 4 and 5 further reduce unique sequence volume by the creation of summary clusters of sequences. See Yu *et al.* (2012) for a detailed explanation of each of the steps. Bracketed values in step 2 represent sequences inferred to be chimeras with the *de novo* chimera detection function UCHIME in USEARCH, all of which were removed in step 4 of PyroClean.

## Discussion

Our approach for the denoising of 454 pyrosequence data from communities of species sampled for protein-coding genes corrects homopolymer error, and filters PCR error, sequencing error, and numts. PyroCleaning of sequence data derived from the amplification of the mtDNA COI gene for two communities of Collembola from forest sites on the island of Tenerife resulted in an average reduction of raw unique sequences to 0.8% within an MID pool. This represents an approximately 16-fold improvement over a comparable alternative approach for denoising protein-coding sequences [15]. We were further able to infer and exclude presumed numts on the basis of their low frequency relative to related mitochondrial genes within an amplicon pool. While this could result in the exclusion of genuine low frequency mitochondrial target genes, studies of broader geographic sampling may help to limit this. Sequence variants that only occur at low frequencies with related higher frequency sequences across multiple sampling sites can more reliably be inferred to be numts. In contrast genuine low frequency mtDNA sequences are not expected to correlate with the distributions and frequencies of related sequences. Thus in Fig. 3, two sequences are inferred to be probable numts, given their low frequency and geographical co-occurrence with sequences of much higher frequency. This inference would gain additional support if further geographic sampling revealed a similar pattern of co-occurrence and frequency difference. .

Our triplicate PCR strategy reveals that independent amplifications from the same template yield broadly similar results with regard to the frequency representation of the template sequences (Table S1). However, it is also clear that these frequencies are not representative of the original template concentrations (Fig. 2). This is likely to be in part explained by our strategy of designing very degenerate primers to improve cross-taxon amplification. Although the pool of primer variants contains at least one theoretically perfect match for every possible template, some template primer pairs will have higher primer binding affinity than others due to such phenomena as differing GC content. Thus, templates with higher GC content at primer binding sites are expected to experience a positive amplification bias. The relationship between read counts and taxon abundance is complex [30], [31], and is also influenced by the extent to which target amplicon copy number correlates with biomass. Estimates of relative abundance have also been demonstrated to be biased for rDNA read counts within microbial communities [30], and we do not advocate extrapolation of qualitative data to quantitative estimation.

PyroClean can yield a sequence dataset that largely removes the need to run time-consuming and sometimes subjective OTU picking procedures. This provides the possibility to characterise fine scale intraspecific biological diversity from PyroCleaned sequence data (e.g. Fig. 3 sequences AE and AF; sequences U, V and W; sequences P, Q and R; sequences B and C), a potentially useful tool for the characterisation of biological communities and their inter-relationships across landscapes [13], which is demonstrated by our analysis of the two Collembolan communities from Tenerife. Collembolan species inhabiting the Canary Islands are all believed to be native, although it is frequently recognised that some species could be the result of introductions [32]. Resolving this issue is complicated by the fact that many species have broad geographic ranges that extend beyond the Canary Islands, but that Canary Island taxa could represent cryptically divergent lineages [13]. We took two approaches to place taxonomic identities to PyroCleaned sequences. The first was by direct comparison to Sanger sequences from taxonomically identified individuals from Tenerife, which resulted in exact sequence matches for 10 PyroCleaned sequences, and taxonomic identities for several lineages that appear to have diversified within Tenerife. Most notable among these are sequences I and J that fall within a monophyletic group of *Friesea* sequences sampled from three other sampling points, suggesting geographically discrete cryptic diversification, an increasingly common observation within Collembola [13]. The second approach to place taxonomic identities with the BOLD Identification System revealed 13 PyroCleaned sequences, representing 8 of the 24 divergent Collembolan lineages, to have 99–100% matching to taxonomically referenced sequences sampled from continental Europe, North America, North Africa and Australia, providing evidence for the contribution of recently introduced species to community composition. Because PyroClean can recover sequences divergent by as little as a single nucleotide, we are able to demonstrate shared genetic diversity between species on Tenerife and populations elsewhere. The three closely related sequences P, Q and R (Fig. 3) are all 100% matches to BOLD database sequences of *Parasitoma notabilis* sampled from Europe, North Africa and Australia, suggesting that some recent introductions of Collembola to Tenerife are of probable human origin.

Many PyroCleaned sequences match to non-collembolan taxa on the BOLD database, which may be explained by (1) PyroClean sequences of non-Collembolan origin, (2) limited Collembolan representation within the BOLD database, or (3) limited taxonomic resolution for the first 220 bases of the barcode gene. Given our sampling protocol, it is unlikely that non-Collembolan tissue would have been amplified unless some species of Collembola ingest animal tissue that could also be amplified by our primers. However, it is not possible to unequivocally rule out sample contamination as a possible explanatory variable. Our data do however suggest that sampling limitations within the BOLD database lead to spurious matching, as seven PyroCleaned lineages, taxonomically identified as Collembola species with referenced Sanger sequences, had closest matches to non-Collembolan taxa on BOLD (Fig. 3, sequences F, I, S, X, Z, AA, AB). PyroClean sequence length also contributes to the issue, as increasing sequence length from 220 bases to the full 658 bases of the barcode region increases taxonomic matching to the Class Collembola (data not shown).

PyroClean extends the suite of existing tools for denoising pyrosequence data (see [14] for a review), facilitating the characterisation of mtDNA COI sequence variation both among and within species, and its spatial structure. Our general approach for the amplification, 454 sequencing, and PyroCleaning of protein-coding DNA sequence data can be applied to any taxonomic group for which prior data exists for generating a consensus reference sequence and primers for amplification. In this context, the mtDNA COI barcoding region is the gene of choice, with the added advantage of a taxonomically and geographically referenced sequence database [23]. However, as interest and effort extends toward the use of mtDNA sequence data for biodiversity analyses with HTP sequencing (e.g. [14], [15], [17]), our results also offer some cautionary advice. Nuclear copies of mtDNA (numts) present a challenge, and if unaccounted for can lead to the overestimation of diversity. PyroClean filters out numts possessing indels or atypical nonsynonymous substitutions, but numts that that are mutationally indistinct from orthologous mtDNA are bioinformatically more challenging. For now, we suggest a conservative approach to their inference and removal, based on low frequency and co-occurrence with phylogenetically related sequences. Further work to automate their detection and removal may be possible as bigger data sets with increased geographic sampling come to hand. A further caution echoes that of Yu et al. [15], who found that many species from their arthropod survey could not be identified even to family level, due to limited sequence representation on public databases. For microfaunal and mesofaunal metazoan groups, such as the Collembola where species diversity is underestimated and thus undocumented at the DNA sequence level [13], taxonomic assignment of PyroCleaned sequences is likely to be even more limited. Both our data and that of Yu *et al.* [15] reveals that in the absence of sufficiently related sequences on public databases, 454 sequences may be assigned to evolutionarily unrelated taxonomic groupings. The caution we advocate is that, in the absence of relevant sequence data within a taxonomic reference database, both taxonomic assignment and the identification of contaminant mtDNA sequences from non-target taxa is complicated. Although we only amplified Collembola, it remains possible that some lineages, in particular those represented by only a few sequences (e.g. Fig. 3, lineage K) could be derived from contaminating tissues or tissues consumed by Collembola. As pyrosequence read lengths increase, and barcode databases grow, taxonomic assignment will likely increase. As we have demonstrated, Sanger sequence reference libraries will also help to associate PyroCleaned sequences to taxonomically described morphospecies.

PyroClean source code is available as Supplementary Software. Data and source code are also available at https://github.com/homonecloco/PyroClean. Data is also deposited in the Dryad repository: http://dx.doi.org/10.5061/dryad.v2k84.

## Supporting Information

Figure S1 **Undetected compensated indels (see main text) from an alignment of PyroCleaned collembolan mtDNA COI sequences.** In each of the three panels compensated indels at the bottom of a panel are evident within the alignment, as they diverge from a related sequences by a sequential run of mismatching nucleotides, facilitating their identification and removal. Dotted lines represent invariant nucleotide sites within the 220 nucleotide alignment.
(DOC)

Figure S2 **Presumed numts from Collembola that are consistent with the consensus reference sequence.** Target mtDNA COI sequences of Collembola morphospecies are in some cases associated with presumed numts - phylogenetically related, low frequency variants of a target sequence. In the two examples below presumed numts after PyroCleaning of mtDNA COI pyrosequence data are highlighted in grey for the collembolan morphospecies *Ceratophysella gibbosa* (left panel) and *Parasitoma notabilis* (right panel).
(DOC)

Table S1 **Percentage representation of the 27 Collembola genomes within each of the 5 genomic pools, and within each of the three MID tag pools derived from each of the 5 genomic pools.**
(DOC)
