# Supplementary figures and images for: PyroClean: Denoising Pyrosequences from Protein-Coding Amplicons for the Recovery of Interspecific and Intraspecific Genetic Variation
Source: PLoS One. 2013 Mar 1;8(3):e57615. doi: 10.1371/journal.pone.0057615 (PMC3585932; doi:10.1371/journal.pone.0057615)

**Figure S1**


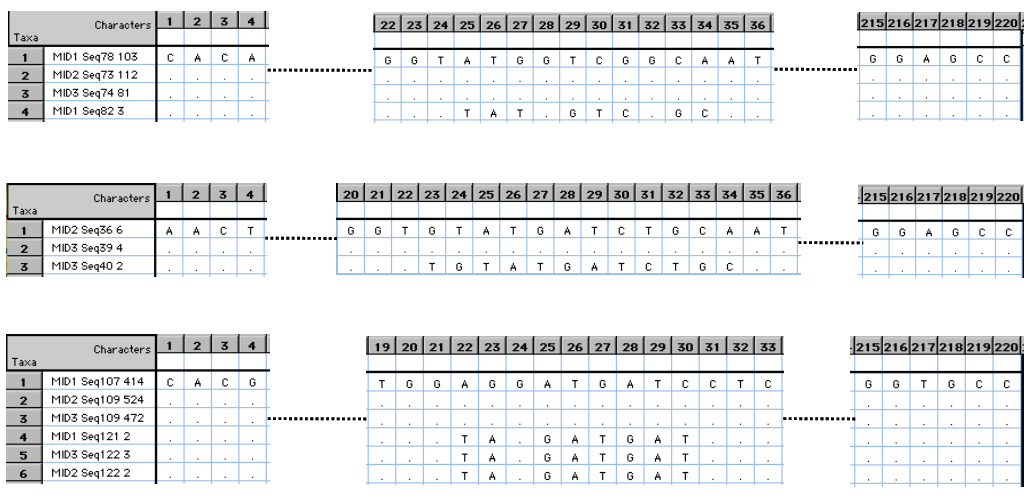

Supplement: Figure S1 — Undetected compensated indels (see main text) from an alignment of PyroCleaned collembolan mtDNA COI sequences. In each of the three panels compensated indels at the bottom of a panel are evident within the alignment, as they diverge from a related sequences by a sequential run of mismatching nucleotides, facilitating their identification and removal. Dotted lines represent invariant nucleotide sites within the 220 nucleotide alignment. (DOC) [file pone.0057615.s001.doc]

**Figure S2**


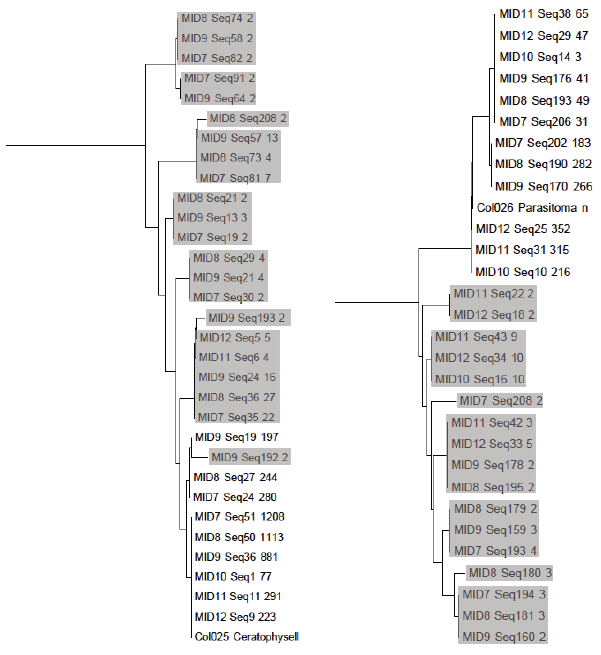

Supplement: Figure S2 — Presumed numts from Collembola that are consistent with the consensus reference sequence. Target mtDNA COI sequences of Collembola morphospecies are in some cases associated with presumed numts - phylogenetically related, low frequency variants of a target sequence. In the two examples below presumed numts after PyroCleaning of mtDNA COI pyrosequence data are highlighted in grey for the collembolan morphospecies Ceratophysella gibbosa (left panel) and Parasitoma notabilis (right panel). (DOC) [file pone.0057615.s002.doc]
