# Supplementary material for: PyroClean: Denoising Pyrosequences from Protein-Coding Amplicons for the Recovery of Interspecific and Intraspecific Genetic Variation
Source: PLoS One. 2013 Mar 1;8(3):e57615. doi: 10.1371/journal.pone.0057615 (PMC3585932; doi:10.1371/journal.pone.0057615)
Supplement: Table S1 — Percentage representation of the 27 Collembola genomes within each of the 5 genomic pools, and within each of the three MID tag pools derived from each of the 5 genomic pools. (DOC) [file pone.0057615.s003.doc]

**Table S1**

|  | **Pool 1** | 1 | MID 2 | 3 | **Pool 2** | 4 | MID 5 | 6 | **Pool 3** | 7 | MID 8 | 9 | **Pool 4** | 10 | MID 11 | 12 | **Pool 5** | 13 | MID 14 | 15 |
| --- | --- | --- | --- | --- | --- | --- | --- | --- | --- | --- | --- | --- | --- | --- | --- | --- | --- | --- | --- | --- |
| Col001 | **7.6** | 18.9 | 22.5 | 20.3 | **10.1** | 31.3 | 31.3 | 28.3 | **5.4** | 7.9 | 15.7 | 14.7 | **23.9** | 39.0 | 43.7 | 50.3 | **0.0** | 0.0 | 0.0 | 0.0 |
| Col002 | **2.8** | 3.4 | 3.4 | 3.0 | **2.2** | 7.0 | 7.7 | 7.1 | **4.0** | 5.6 | 5.7 | 4.2 | **0.0** | 0.0 | 0.0 | 0.0 | **8.3** | 14.0 | 13.7 | 12.3 |
| Col005 | **2.1** | 0.0 | 0.2 | 0.2 | **1.1** | 0.0 | 0.1 | 0.2 | **3.0** | 0.3 | 0.4 | 0.5 | **0.0** | 0.0 | 0.0 | 0.0 | **6.3** | 0.5 | 0.5 | 0.6 |
| Col023 | **4.9** | 4.8 | 4.6 | 4.4 | **3.9** | 3.5 | 4.0 | 3.5 | **3.5** | 3.0 | 2.8 | 3.3 | **0.0** | 0.0 | 0.0 | 0.0 | **0.0** | 0.0 | 0.0 | 0.0 |
| Col024 | **1.5** | 1.1 | 1.6 | 1.1 | **0.8** | 1.6 | 1.0 | 0.9 | **2.2** | 1.9 | 2.6 | 3.3 | **0.0** | 0.0 | 0.0 | 0.0 | **0.0** | 0.0 | 0.0 | 0.0 |
| Col025 | **0.5** | 1.5 | 1.1 | 1.9 | **0.1** | 0.3 | 0.3 | 0.5 | **1.8** | 5.5 | 5.4 | 4.2 | **0.8** | 1.3 | 2.9 | 1.3 | **0.0** | 0.0 | 0.0 | 0.0 |
| Col026 | **0.9** | 2.2 | 1.4 | 2.1 | **0.5** | 1.1 | 0.6 | 0.7 | **2.0** | 4.7 | 3.6 | 3.9 | **0.0** | 0.0 | 0.0 | 0.0 | **1.9** | 6.5 | 7.0 | 7.7 |
| Col027 | **1.0** | 0.1 | 0.0 | 0.2 | **0.5** | 0.1 | 0.2 | 0.0 | **2.2** | 0.0 | 0.1 | 0.4 | **0.0** | 0.0 | 0.0 | 0.0 | **0.0** | 0.0 | 0.0 | 0.0 |
| Col034 | **0.6** | 0.3 | 0.2 | 0.3 | **0.2** | 0.0 | 0.1 | 0.0 | **2.3** | 0.9 | 1.0 | 0.7 | **0.0** | 0.0 | 0.0 | 0.0 | **0.6** | 0.2 | 0.2 | 0.4 |
| Col038 | **5.4** | 3.6 | 3.5 | 3.0 | **3.9** | 2.4 | 3.0 | 2.6 | **5.1** | 5.6 | 6.5 | 6.7 | **17.7** | 10.2 | 11.8 | 10.4 | **0.0** | 0.0 | 0.0 | 0.0 |
| Col042 | **15.8** | 2.8 | 2.7 | 3.2 | **17.8** | 2.2 | 2.8 | 2.6 | **12.3** | 2.5 | 2.6 | 2.3 | **35.0** | 4.7 | 5.3 | 4.0 | **12.1** | 8.1 | 6.0 | 6.2 |
| Col052 | **9.5** | 3.7 | 4.6 | 4.9 | **12.7** | 6.5 | 6.7 | 6.9 | **6.8** | 3.2 | 2.9 | 2.6 | **0.0** | 0.0 | 0.0 | 0.0 | **0.0** | 0.0 | 0.0 | 0.0 |
| Col054 | **12.6** | 14.6 | 10.0 | 13.1 | **16.7** | 15.4 | 13.6 | 15.9 | **9.0** | 9.4 | 7.0 | 7.2 | **0.0** | 0.0 | 0.1 | 0.0 | **12.5** | 19.0 | 20.0 | 19.6 |
| Col055 | **9.8** | 1.3 | 1.4 | 1.9 | **13.0** | 1.3 | 1.5 | 1.3 | **7.0** | 1.8 | 0.5 | 0.3 | **0.0** | 0.0 | 0.3 | 0.1 | **19.6** | 6.7 | 6.3 | 7.2 |
| Col056 | **2.5** | 2.4 | 2.5 | 2.6 | **2.0** | 1.6 | 2.0 | 2.3 | **3.5** | 3.7 | 3.8 | 5.1 | **11.7** | 8.1 | 9.3 | 8.8 | **0.0** | 0.0 | 0.0 | 0.0 |
| Col057 | **0.5** | 2.7 | 1.8 | 2.1 | **0.1** | 1.1 | 0.8 | 1.2 | **1.8** | 12.4 | 11.9 | 10.3 | **0.0** | 0.0 | 0.0 | 0.0 | **0.5** | 3.7 | 4.2 | 3.3 |
| Col058 | **3.0** | 16.1 | 17.4 | 14.1 | **2.4** | 11.1 | 11.1 | 13.5 | **0.0** | 0.0 | 0.0 | 0.0 | **0.0** | 0.0 | 0.0 | 0.0 | **0.0** | 0.0 | 0.0 | 0.0 |
| Col059 | **0.7** | 1.3 | 1.4 | 1.9 | **0.2** | 1.3 | 1.5 | 1.3 | **2.7** | 1.8 | 0.5 | 0.3 | **1.2** | 0.0 | 0.3 | 0.1 | **0.7** | 6.7 | 6.3 | 7.2 |
| Col060 | **3.2** | 0.4 | 0.5 | 0.4 | **2.6** | 0.3 | 0.2 | 0.1 | **4.6** | 0.5 | 0.6 | 0.3 | **0.0** | 0.0 | 0.0 | 0.0 | **13.0** | 2.8 | 2.8 | 1.9 |
| Col061 | **0.6** | 1.1 | 1.4 | 1.2 | **0.2** | 0.7 | 0.4 | 0.4 | **2.1** | 4.6 | 5.6 | 6.3 | **0.0** | 0.0 | 0.1 | 0.0 | **0.6** | 2.6 | 2.9 | 3.9 |
| Col063 | **1.9** | 0.6 | 0.5 | 0.4 | **1.0** | 0.3 | 0.2 | 0.1 | **2.7** | 0.5 | 0.5 | 0.4 | **0.0** | 0.0 | 0.0 | 0.0 | **3.7** | 1.4 | 1.7 | 1.4 |
| Col068 | **5.3** | 4.6 | 4.6 | 3.4 | **4.3** | 3.0 | 4.3 | 3.8 | **3.8** | 3.9 | 2.7 | 4.2 | **0.0** | 0.0 | 0.0 | 0.0 | **16.0** | 18.7 | 20.4 | 19.4 |
| Col070 | **1.1** | 4.9 | 4.6 | 4.4 | **0.6** | 3.2 | 2.0 | 2.4 | **2.3** | 8.7 | 7.0 | 7.8 | **0.0** | 0.0 | 0.0 | 0.0 | **1.1** | 8.4 | 7.3 | 8.4 |
| Col072 | **1.9** | 6.6 | 7.0 | 8.4 | **1.0** | 3.8 | 4.1 | 3.8 | **2.7** | 10.2 | 8.5 | 8.4 | **8.8** | 36.5 | 25.6 | 24.4 | **0.0** | 0.0 | 0.0 | 0.0 |
| Col075 | **0.5** | 0.3 | 0.5 | 0.2 | **0.1** | 0.2 | 0.1 | 0.2 | **1.9** | 0.9 | 1.1 | 1.3 | **0.9** | 0.2 | 0.5 | 0.5 | **0.0** | 0.0 | 0.0 | 0.0 |
| Col080 | **1.5** | 0.3 | 0.3 | 0.7 | **0.8** | 0.3 | 0.2 | 0.3 | **2.2** | 0.4 | 0.7 | 0.9 | **0.0** | 0.0 | 0.0 | 0.0 | **3.0** | 0.8 | 0.6 | 0.5 |
| Col082 | **2.2** | 0.3 | 0.2 | 0.5 | **1.2** | 0.4 | 0.2 | 0.2 | **3.2** | 0.3 | 0.4 | 0.3 | **0.0** | 0.0 | 0.0 | 0.0 | **0.0** | 0.0 | 0.0 | 0.0 |

Taxonomic identities. Col001: *Latryopiga longiseta*, Col002: *Protaphorura armata*, Col005: *Heteromurus nitidis*, Col023: *Lepidocyrtus* cf *cyaneus*, Col024: *Sminthurinus elegans*, Col025: *Ceratophysella gibbosa*, Col026: *Parisitoma notabilis*, Col027: *Deutaphorura* sp., Col034: *Entomobrya atrocinta*, Col038: *Lepidocyrtus cyaneus*, Col042: *Lepidocyrtus lignorum*, Col052: *Neanura muscorum*, Col054: *Tomocerus vulgaris*, Col055: *Entomobrya nicoleti*, Col056: *Pseudosinella alba*, Col057: *Sminthurinus elegans*, Col058: *Isotoma viridis* group, Col059: *Entomobrya intermedia*, Col060: *Lepidocyrtus cyaneus*, Col061: *Parisotoma notabilis*, Col063: *Heteromurus nitidis*, Col068: *Lepidocyrtus* sp., Col070: *Hypogastrura socialis*, Col072: *Vertagopus arborea*, Col075: *Bilobella aurantiaca*, Col080: *Lepidocyrtus lignorum*, Col082: *Heteromurus major*.
